# Supplementary material for: Lactobacillus reuteri Colonisation of Extremely Preterm Infants in a Randomised Placebo-Controlled Trial
Source: Microorganisms. 2021 Apr 24;9(5):915. doi: 10.3390/microorganisms9050915 (PMC8190634; doi:10.3390/microorganisms9050915)
Supplement: Supplementary file 1 [file microorganisms-09-00915-s001.zip › microorganisms-1184656-SI.pdf]

Supplementary material

Supplementary figures

Figure S1: Maternal and infant characteristics and *L. reuteri* abundance in faeces of the *L. reuteri*-supplemented group at one week of age

Figure S2: Maternal and infant characteristics and *L. reuteri* abundance in faeces of the *L. reuteri*-supplemented group at PMW 36

Figure S3: Length and head circumference growth by supplementation group and sex

Supplementary tables

Table S1: Human milk oligosaccharides and a mother's Lewis and secretor status

Table S2: Background characteristics of extremely preterm ELBW infants in the *L. reuteri*-supplemented and placebo group

Table S3: Maternal and infant characteristics and *L. reuteri* abundance in the *L. reuteri*-supplemented group at one week of age

Table S4: Antibiotic treatment and *L. reuteri* prevalence in faeces of the *L. reuteri*-supplemented group

Table S5: Antibiotic treatment and *L. reuteri* abundance in faeces of the *L. reuteri*-supplemented group

Table S6: Maternal Lewis and secretor status and *L. reuteri* colonisation in the *L. reuteri*-supplemented group

Table S7: Human milk oligosaccharides and *L. reuteri* prevalence in the *L. reuteri*-supplemented group

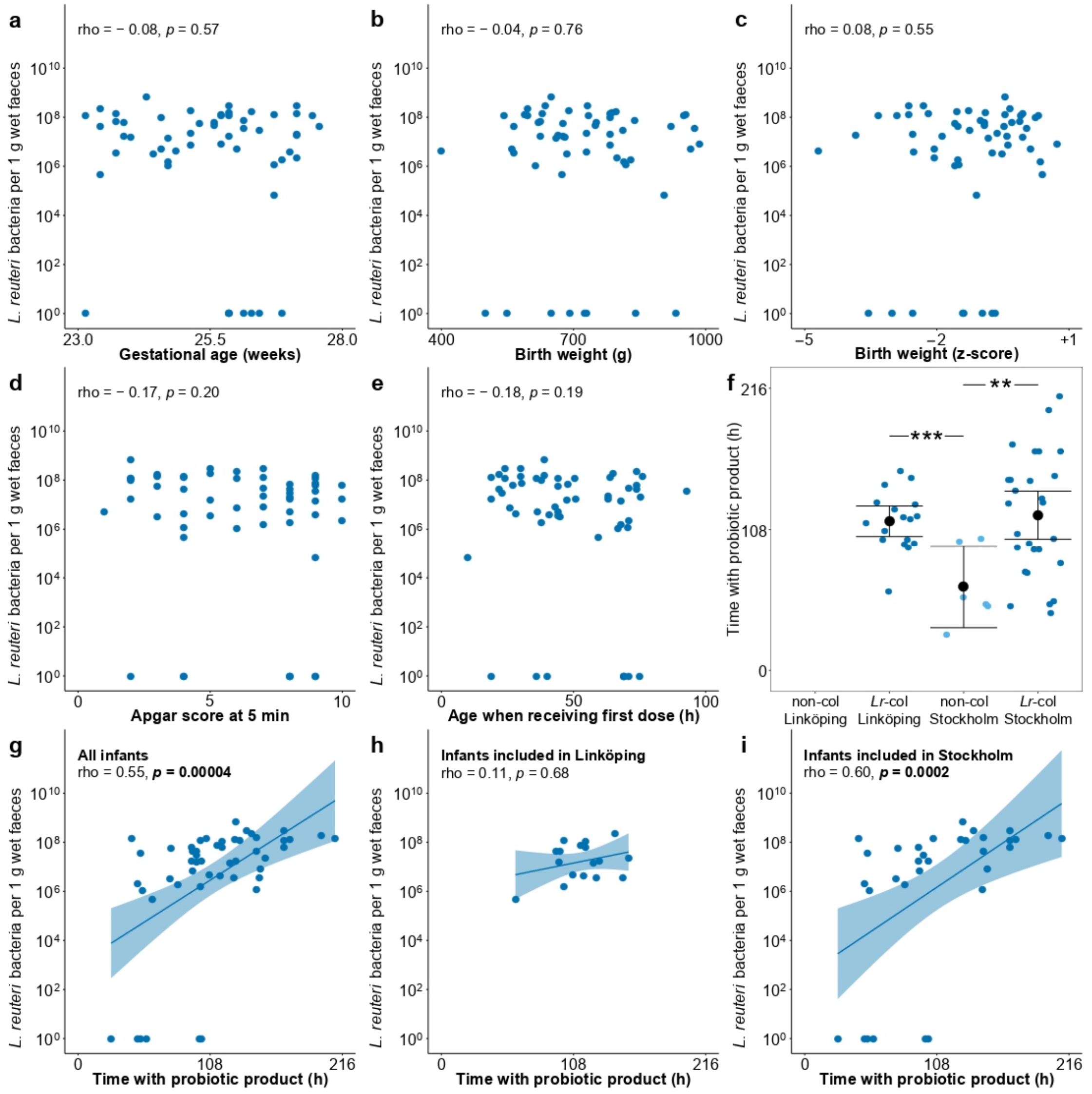

Figure S1: Maternal and infant characteristics and *L. reuteri* abundance in faeces of the *L. reuteri*-supplemented group at one week of age. (**a-e**) Spearman correlation between the gestational age (in weeks) (**a**), the birth weight (in grams) (**b**), the birth weight (z-score) (**c**), the Apgar score at 5 min (**d**), the age when receiving the first dose of *L. reuteri* (in hours) (**e**) and *L. reuteri* abundance in faeces of *L. reuteri*-supplemented infants at one week of age. (**f**) Total time with the probiotic product before faecal sampling at one week of age (in hours) in non-colonised (non-col) and *L. reuteri*-colonised (*Lr*-col) infants included in Linköping and Stockholm. Black dot represents the mean and error bars the 95% confidence interval. (**g-i**) Spearman correlation between the total time with the probiotic product before faecal sampling at one week of age (in hours) in all *L. reuteri*-supplemented infants (**g**), and in *L. reuteri*-supplemented infants included in Linköping (**h**) and Stockholm (**i**) and *L. reuteri* abundance in faeces of *L. reuteri*-supplemented infants at one week of age. Abundance is expressed as *L. reuteri* bacteria per 1 g wet faeces. For infants with a faecal sample negative for *L. reuteri*, the number of *L. reuteri* bacteria per 1 g wet faeces was set to 10^0. For two infants included in Linköping and four infants included in Stockholm, the total time with the probiotic product

was not known. Statistics: Spearman correlation (**a-e, g-i**). Student's *t*-test (**f**). ** $p < 0.01$, *** $p < 0.001$.

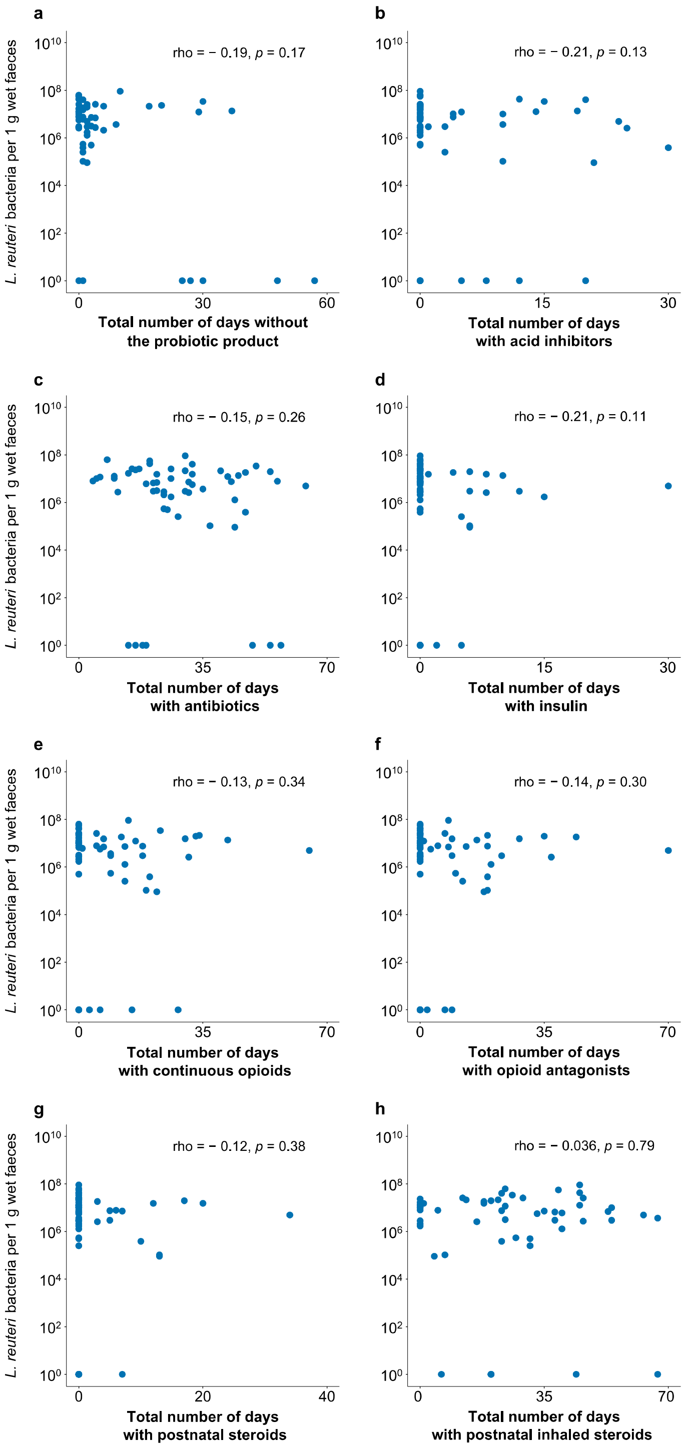

Figure S2: Maternal and infant characteristics and *L. reuteri* abundance in faeces of the *L. reuteri*-supplemented group at PMW 36. Spearman correlation between the total number of days (from birth until PMW 36) without the probiotic product (**a**), with acid inhibitors (**b**), with antibiotics (**c**), with insulin (**d**), with continuous opioids (**e**), with opioid antagonists (**f**), with postnatal steroids (**g**), and with postnatal inhaled steroids (**h**) and *L. reuteri* abundance at PMW 36. Abundance is expressed as *L. reuteri* bacteria per 1 g wet faeces. For infants with a faecal sample negative for *L. reuteri*, the number of *L. reuteri* bacteria per 1 g wet faeces was

set to 10^0. For one infant, the total number of days with postnatal inhaled steroids was not known (**h**). PMW 36 = postmenstrual week 36.

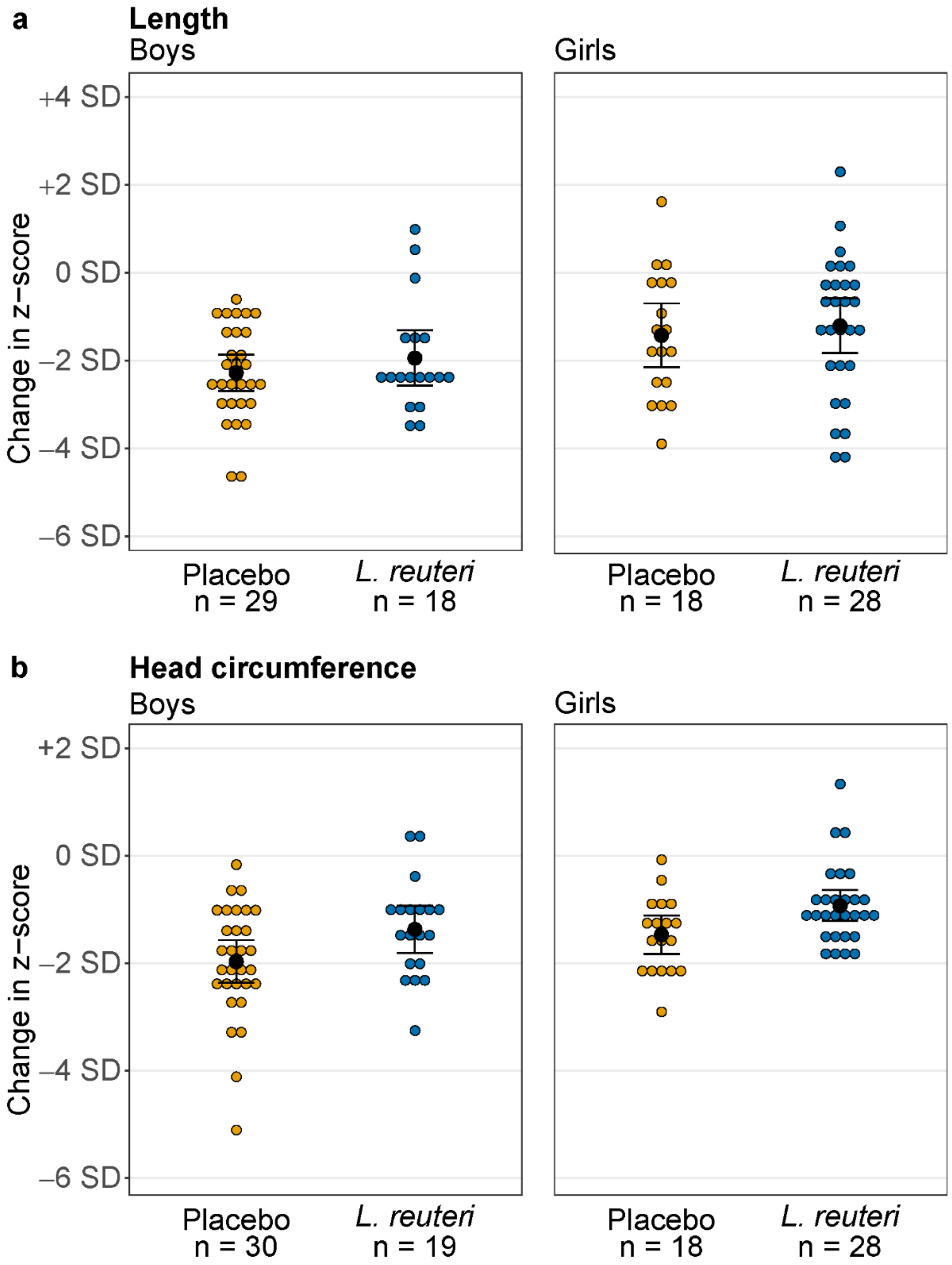

Figure S3: Length and head circumference growth by supplementation group and sex. Changes in z-score for length (**a**) and head circumference (**b**) growth from birth to four weeks of age in placebo and *L. reuteri*-supplemented infants with a faecal sample negative or positive for *L. reuteri* at three weeks of age, respectively. Infants are stratified by sex. Coloured dots represent the change in z-score from birth to four weeks of age for individual infants. Black dots represent group means and error bars represent 95% confidence intervals. Length growth data for one infant of each group are missing.

Table S1: Human milk oligosaccharides and a mother's Lewis and secretor status. The table shows the 15 major oligosaccharides that were measured in human milk. The column named 'Secreted by' indicates whether an oligosaccharide is expected in milk based on a mother's Lewis (Le) and secretor (Se) status. Le+ = Lewis-positive, Se+ = Secretor-positive.

**Human milk oligosaccharide**		**Secreted by**
Disialyl-lacto-N-tetraose	(DSLNT)	All
2′-Fucosyllactose	(2′FL)	Se+
3′-Fucosyllactose	(3′FL)	All
Lacto-difucotetraose	(LDFT)	Se+
Lacto-N-difucohexaose I	(LNDH I)	Le+ Se+
Lacto-N-fucopentaose I	(LNFP I)	Se+
Lacto-N-fucopentaose II	(LNFP II)	Le+
Lacto-N-fucopentaose III	(LNFP III)	All
Lacto-N-neotetraose	(LNnT)	All
Lacto-N-tetraose	(LNT)	All
Sialyl-lacto-N-tetraose a	(LSTa)	All
Sialyl-lacto-N-tetraose b	(LSTb)	All
Sialyl-lacto-N-neotetraose c	(LSTc)	All
3′-Sialyllactose	(3′SL)	All
6′-Sialyllactose	(6′SL)	All

Table S2: Background characteristics of extremely preterm ELBW infants in the *L. reuteri*-supplemented and placebo group.

Background and clinical characteristics of *L. reuteri*- and placebo-supplemented infants for whom faecal samples were available at one (**a**), two (**b**), three (**c**), and four weeks of age (**d**), at postmenstrual week 36 (**e**), and at two years of age (**f**), respectively. ELBW = extremely low birth weight.

**a: Background characteristics of infants with a faecal sample at one week of age**					
	**Placebo (*n* = 57)**		***L. reuteri* (*n* = 56)**		***p* value**
Gestational age, weeks, mean (SD)	25.6	(1.2)	25.5	(1.2)	0.75[1]
Gestational weeks 23-25, n (%)	32	(56%)	35	(62%)	0.57[2]
Birth weight, g, mean (SD)	754	(143)	724	(131)	0.25[1]
Birth weight, z-score, mean (SD)	-1.1	(1.4)	-1.3	(1.2)	0.51[1]
Birth length, cm, mean (SD)	32.9	(2.6)	32.3	(2.5)	0.23[1]
Birth length, z-score, mean (SD)	-1.3	(1.9)	-1.8	(1.7)	0.24[1]
Birth head circumference, cm, mean (SD)	23.3	(1.6)	22.9	(1.4)	0.13[1]
Birth head circumference, z-score, mean (SD)	-0.7	(0.8)	-1.0	(0.8)	0.09[1]
Apgar score at 1 min, median (IQR)[a,b]	5.0	(3.8 - 6.0)	5.0	(3.0 - 6.5)	0.73[3]
Apgar score at 5 min, median (IQR)[a]	7.0	(5.8 - 8.0)	7.0	(4.0 - 8.0)	0.42[3]
Apgar score at 10 min, median (IQR)[a]	8.0	(7.0 - 9.2)	8.0	(6.8 - 9.0)	0.23[3]
Small for gestational age (weight < 2 SD), n (%)	12	(21%)	17	(30%)	0.29[2]
Male/Female, n/n (%/%)	37/20	(65%/35%)	24/32	(43%/57%)	**0.02**[2]
Infant from multiple pregnancy, n (%)	18	(32%)	19	(34%)	0.84[2]
Caesarean section, n (%)	33	(58%)	42	(75%)	0.07[2]
Chorioamnionitis, n (%)	8	(14%)	16	(29%)	0.07[2]
Preeclampsia, n (%)	4	(7%)	5	(9%)	0.74[2]
Preterm premature rupture of membranes, n (%)	14	(25%)	21	(38%)	0.16[2]
Maternal smoking, n (%)	5	(9%)	4	(7%)	1.00[2]
Maternal antibiotics, n (%)	28	(49%)	35	(62%)	0.19[2]
Intubated at inclusion, n (%)	41	(72%)	47	(84%)	0.17[2]
Surfactant, n (%)	43	(75%)	48	(86%)	0.23[2]
Antenatal corticosteroids, n (%)	56	(98%)	55	(98%)	1.00[2]
Full course of antenatal corticosteroids, n (%)	40	(70%)	39	(70%)	1.00[2]
Patent ductus arteriosus, n (%)	40	(70%)	40	(71%)	1.00[2]
Surgical ligation of ductus arteriosus, n (%)	17	(30%)	20	(36%)	0.55[2]
Inclusion site - Linköping/Stockholm, n/n (%/%)	20/37	(35%/65%)	19/37	(34%/66%)	1.00[2]

[a]Data for one child from the placebo group are missing. [b]Data for one child from the *L. reuteri*-supplemented group are missing. Statistics: [1]Student's *t*-test, [2]Fisher's exact test, [3]Mann-Whitney *U* test. IQR = interquartile range, SD = standard deviation.

b: Background characteristics of infants with a faecal sample at two weeks of age					
	**Placebo (*n* = 56)**		***L. reuteri* (*n* = 55)**		***p* value**
Gestational age, weeks, mean (SD)	25.5	(1.3)	25.5	(1.3)	0.95[1]
Gestational weeks 23-25, n (%)	32	(57%)	34	(62%)	0.70[2]
Birth weight, g, mean (SD)	750	(145)	732	(133)	0.49[1]
Birth weight, z-score, mean (SD)	-1.1	(1.4)	-1.2	(1.2)	0.54[1]
Birth length, cm, mean (SD)	32.8	(2.6)	32.3	(2.5)	0.33[1]
Birth length, z-score, mean (SD)	-1.3	(1.9)	-1.7	(1.8)	0.25[1]
Birth head circumference, cm, mean (SD)	23.2	(1.7)	23.0	(1.4)	0.43[1]
Birth head circumference, z-score, mean (SD)	-0.7	(0.8)	-0.9	(0.8)	0.20[1]
Apgar score at 1 min, median (IQR)[a,b]	5.0	(2.5 - 6.0)	5.0	(4.0 - 6.0)	0.42[3]
Apgar score at 5 min, median (IQR)[a]	6.0	(5.0 - 8.0)	7.0	(4.0 - 8.0)	0.80[3]
Apgar score at 10 min, median (IQR)[a]	8.0	(7.0 - 9.0)	8.0	(7.0 - 9.0)	0.86[3]
Small for gestational age (weight < 2 SD), n (%)	11	(20%)	17	(31%)	0.20[2]
Male/Female, n/n (%/%)	36/20	(64%/36%)	25/30	(45%/55%)	0.06[2]
Infant from multiple pregnancy, n (%)	18	(32%)	24	(44%)	0.24[2]
Caesarean section, n (%)	32	(57%)	42	(76%)	**0.04**[2]
Chorioamnionitis, n (%)	10	(18%)	16	(29%)	0.18[2]
Preeclampsia, n (%)	4	(7%)	5	(9%)	0.74[2]
Preterm premature rupture of membranes, n (%)	14	(25%)	18	(33%)	0.41[2]
Maternal smoking, n (%)	4	(7%)	4	(7%)	1.00[2]
Maternal antibiotics, n (%)	30	(54%)	32	(58%)	0.70[2]
Intubated at inclusion, n (%)	41	(73%)	46	(84%)	0.25[2]
Surfactant, n (%)	43	(77%)	48	(87%)	0.22[2]
Antenatal corticosteroids, n (%)	55	(98%)	54	(98%)	1.00[2]
Full course of antenatal corticosteroids, n (%)	39	(70%)	39	(71%)	1.00[2]
Patent ductus arteriosus, n (%)	41	(73%)	40	(73%)	1.00[2]
Surgical ligation of ductus arteriosus, n (%)	18	(32%)	20	(36%)	0.69[2]
Inclusion site - Linköping/Stockholm, n/n (%/%)	21/35	(38%/62%)	19/36	(35%/65%)	0.84[2]

[a]Data for one child from the placebo group are missing. [b]Data for one child from the *L. reuteri*-supplemented group are missing. Statistics: [1]Student's *t*-test, [2]Fisher's exact test, [3]Mann-Whitney *U* test. IQR = interquartile range, SD = standard deviation.

**c: Background characteristics of infants with a faecal sample at three weeks of age**					
	**Placebo (*n* = 53)**		***L. reuteri* (*n* = 51)**		***p* value**
Gestational age, weeks, mean (SD)	25.6	(1.2)	25.6	(1.2)	0.86[1]
Gestational weeks 23-25, n (%)	29	(55%)	31	(61%)	0.56[2]
Birth weight, g, mean (SD)	749	(145)	736	(122)	0.62[1]
Birth weight, z-score, mean (SD)	-1.1	(1.4)	-1.2	(1.2)	0.66[1]
Birth length, cm, mean (SD)	32.9	(2.6)	32.6	(2.4)	0.54[1]
Birth length, z-score, mean (SD)	-1.3	(1.9)	-1.6	(1.6)	0.39[1]
Birth head circumference, cm, mean (SD)	23.4	(1.7)	23.0	(1.3)	0.28[1]
Birth head circumference, z-score, mean (SD)	-0.7	(0.8)	-0.9	(0.8)	0.08[1]
Apgar score at 1 min, median (IQR)[a,b]	5.0	(3.0 - 6.0)	5.0	(4.0 - 6.8)	0.29[3]
Apgar score at 5 min, median (IQR)[a]	7.0	(5.0 - 8.0)	7.0	(4.0 - 8.0)	0.76[3]
Apgar score at 10 min, median (IQR)[a]	8.0	(7.0 - 9.0)	8.0	(7.0 - 9.0)	0.49[3]
Small for gestational age (weight < 2 SD), n (%)	11	(21%)	16	(31%)	0.27[2]
Male/Female, n/n (%/%)	34/19	(64%/36%)	22/29	(43%/57%)	**0.049**[2]
Infant from multiple pregnancy, n (%)	18	(34%)	23	(45%)	0.32[2]
Caesarean section, n (%)	31	(58%)	42	(82%)	**0.01**[2]
Chorioamnionitis, n (%)	8	(15%)	16	(31%)	0.06[2]
Preeclampsia, n (%)	5	(9%)	5	(10%)	1.00[2]
Preterm premature rupture of membranes, n (%)	13	(25%)	17	(33%)	0.39[2]
Maternal smoking, n (%)	4	(8%)	3	(6%)	1.00[2]
Maternal antibiotics, n (%)	26	(49%)	32	(63%)	0.17[2]
Intubated at inclusion, n (%)	39	(74%)	40	(78%)	0.65[2]
Surfactant, n (%)	41	(77%)	42	(82%)	0.63[2]
Antenatal corticosteroids, n (%)	53	(100%)	50	(98%)	0.49[2]
Full course of antenatal corticosteroids, n (%)	38	(72%)	34	(67%)	0.67[2]
Patent ductus arteriosus, n (%)	41	(77%)	38	(75%)	0.82[2]
Surgical ligation of ductus arteriosus, n (%)	17	(32%)	19	(37%)	0.68[2]
Inclusion site - Linköping/Stockholm, n/n (%/%)	21/32	(40%/60%)	18/33	(35%/65%)	0.69[2]

[a]Data for one child from the placebo group are missing. [b]Data for one child from the *L. reuteri*-supplemented group are missing. Statistics: [1]Student's *t*-test, [2]Fisher's exact test, [3]Mann-Whitney *U* test. IQR = interquartile range, SD = standard deviation.

d: Background characteristics of infants with a faecal sample at four weeks of age					
	**Placebo (*n* = 51)**		***L. reuteri* (*n* = 53)**		***p* value**
Gestational age, weeks, mean (SD)	25.5	(1.3)	25.5	(1.1)	0.89[1]
Gestational weeks 23-25, n (%)	29	(57%)	34	(64%)	0.55[2]
Birth weight, g, mean (SD)	745	(141)	733	(128)	0.65[1]
Birth weight, z-score, mean (SD)	-1.1	(1.4)	-1.2	(1.2)	0.67[1]
Birth length, cm, mean (SD)	32.8	(2.6)	32.5	(2.5)	0.63[1]
Birth length, z-score, mean (SD)	-1.4	(1.9)	-1.6	(1.7)	0.54[1]
Birth head circumference, cm, mean (SD)	23.3	(1.7)	23.0	(1.4)	0.34[1]
Birth head circumference, z-score, mean (SD)	-0.7	(0.8)	-1.0	(0.9)	0.14[1]
Apgar score at 1 min, median (IQR)[a,b]	5.0	(2.0 - 6.0)	5.0	(3.8 - 7.0)	0.14[3]
Apgar score at 5 min, median (IQR)[a]	7.0	(5.0 - 8.0)	7.0	(4.0 - 8.0)	0.98[3]
Apgar score at 10 min, median (IQR)[a]	8.0	(7.0 - 9.0)	8.0	(7.0 - 9.0)	0.70[3]
Small for gestational age (weight < 2 SD), n (%)	11	(22%)	18	(34%)	0.19[2]
Male/Female, n/n (%/%)	33/18	(65%/35%)	26/27	(49%/51%)	0.12[2]
Infant from multiple pregnancy, n (%)	15	(29%)	21	(40%)	0.31[2]
Caesarean section, n (%)	30	(59%)	41	(77%)	0.06[2]
Chorioamnionitis, n (%)	7	(14%)	15	(28%)	0.09[2]
Preeclampsia, n (%)	6	(12%)	5	(9%)	0.76[2]
Preterm premature rupture of membranes, n (%)	11	(22%)	19	(36%)	0.13[2]
Maternal smoking, n (%)	5	(10%)	4	(8%)	0.74[2]
Maternal antibiotics, n (%)	25	(49%)	34	(64%)	0.17[2]
Intubated at inclusion, n (%)	37	(73%)	42	(79%)	0.49[2]
Surfactant, n (%)	39	(76%)	43	(81%)	0.63[2]
Antenatal corticosteroids, n (%)	50	(98%)	53	(100%)	0.49[2]
Full course of antenatal corticosteroids, n (%)	35	(69%)	37	(70%)	1.00[2]
Patent ductus arteriosus, n (%)	38	(75%)	39	(74%)	1.00[2]
Surgical ligation of ductus arteriosus, n (%)	17	(33%)	20	(38%)	0.69[2]
Inclusion site - Linköping/Stockholm, n/n (%/%)	20/31	(39%/61%)	18/35	(34%/66%)	0.68[2]

[a]Data for one child from the placebo group are missing. [b]Data for one child from the *L. reuteri*-supplemented group are missing. Statistics: [1]Student's *t*-test, [2]Fisher's exact test, [3]Mann-Whitney *U* test. IQR = interquartile range, SD = standard deviation.

e: Background characteristics of infants with a faecal sample at postmenstrual week 36					
	**Placebo (*n* = 47)**		***L. reuteri* (*n* = 56)**		***p* value**
Gestational age, weeks, mean (SD)	25.5	(1.1)	25.5	(1.2)	0.88[1]
Gestational weeks 23-25, n (%)	28	(60%)	35	(62%)	0.84[2]
Birth weight, g, mean (SD)	749	(142)	742	(124)	0.81[1]
Birth weight, z-score, mean (SD)	-1.1	(1.5)	-1.1	(1.1)	0.87[1]
Birth length, cm, mean (SD)	32.9	(2.4)	32.5	(2.3)	0.43[1]
Birth length, z-score, mean (SD)	-1.3	(1.9)	-1.6	(1.6)	0.35[1]
Birth head circumference, cm, mean (SD)	23.3	(1.6)	23.1	(1.3)	0.48[1]
Birth head circumference, z-score, mean (SD)	-0.7	(0.8)	-0.9	(0.8)	0.24[1]
Apgar score at 1 min, median (IQR)[a,b]	5.0	(3.0 - 6.0)	5.0	(4.0 - 7.0)	0.32[3]
Apgar score at 5 min, median (IQR)[a]	6.0	(5.0 - 8.0)	7.0	(4.0 - 9.0)	1.00[3]
Apgar score at 10 min, median (IQR)[a]	8.0	(7.0 - 9.8)	8.0	(7.0 - 9.0)	0.69[3]
Small for gestational age (weight < 2 SD), n (%)	11	(23%)	16	(29%)	0.65[2]
Male/Female, n/n (%/%)	32/15	(68%/32%)	27/29	(48%/52%)	**0.048**[2]
Infant from multiple pregnancy, n (%)	11	(23%)	26	(46%)	**0.02**[2]
Caesarean section, n (%)	24	(51%)	43	(77%)	**0.01**[2]
Chorioamnionitis, n (%)	6	(13%)	19	(34%)	**0.02**[2]
Preeclampsia, n (%)	4	(9%)	6	(11%)	0.75[2]
Preterm premature rupture of membranes, n (%)	10	(21%)	21	(38%)	0.09[2]
Maternal smoking, n (%)	3	(6%)	4	(7%)	1.00[2]
Maternal antibiotics, n (%)	23	(49%)	37	(66%)	0.11[2]
Intubated at inclusion, n (%)	33	(70%)	43	(77%)	0.50[2]
Surfactant, n (%)	35	(74%)	45	(80%)	0.49[2]
Antenatal corticosteroids, n (%)	46	(98%)	56	(100%)	0.46[2]
Full course of antenatal corticosteroids, n (%)	34	(72%)	41	(73%)	1.00[2]
Patent ductus arteriosus, n (%)	34	(72%)	39	(70%)	0.83[2]
Surgical ligation of ductus arteriosus, n (%)	16	(34%)	19	(34%)	1.00[2]
Inclusion site - Linköping/Stockholm, n/n (%/%)	20/27	(43%/57%)	18/38	(32%/68%)	0.31[2]

[a]Data for one child from the placebo group are missing. [b]Data for one child from the *L. reuteri*-supplemented group are missing. Statistics: [1]Student's *t*-test, [2]Fisher's exact test, [3]Mann-Whitney *U* test. IQR = interquartile range, SD = standard deviation.

f: Background characteristics of infants with a faecal sample at two years of age					
	**Placebo (*n* = 27)**		***L. reuteri* (*n* = 20)**		***p* value**
Gestational age, weeks, mean (SD)	25.3	(1.3)	25.4	(1.2)	0.80[1]
Gestational weeks 23-25, n (%)	17	(63%)	14	(70%)	0.76[2]
Birth weight, g, mean (SD)	727	(152)	736	(113)	0.82[1]
Birth weight, z-score, mean (SD)	-1.1	(1.2)	-1.0	(1.0)	0.87[1]
Birth length, cm, mean (SD)	32.7	(2.7)	32.9	(2.4)	0.75[1]
Birth length, z-score, mean (SD)	-1.2	(1.8)	-1.1	(1.4)	0.86[1]
Birth head circumference, cm, mean (SD)	23.2	(2.0)	23.1	(1.5)	0.81[1]
Birth head circumference, z-score, mean (SD)	-0.6	(0.8)	-0.8	(0.8)	0.36[1]
Apgar score at 1 min, median (IQR)	5.0	(2.0 - 6.0)	4.0	(4.0 - 7.0)	0.29[3]
Apgar score at 5 min, median (IQR)	6.0	(5.0 - 7.0)	7.0	(4.0 - 9.0)	0.51[3]
Apgar score at 10 min, median (IQR)	8.0	(7.0 - 9.0)	8.0	(7.0 - 9.0)	0.31[3]
Small for gestational age (weight < 2 SD), n (%)	4	(15%)	4	(20%)	0.71[2]
Male/Female, n/n (%/%)	17/10	(63%/37%)	10/10	(50%/50%)	0.55[2]
Infant from multiple pregnancy, n (%)	12	(44%)	8	(40%)	1.00[2]
Caesarean section, n (%)	14	(52%)	14	(70%)	0.24[2]
Chorioamnionitis, n (%)	2	(7%)	7	(35%)	**0.03**[2]
Preeclampsia, n (%)	3	(11%)	0	(0%)	0.25[2]
Preterm premature rupture of membranes, n (%)	6	(22%)	6	(30%)	0.74[2]
Maternal smoking, n (%)	3	(11%)	0	(0%)	0.25[2]
Maternal antibiotics, n (%)	14	(52%)	13	(65%)	0.39[2]
Intubated at inclusion, n (%)	22	(81%)	16	(80%)	1.00[2]
Surfactant, n (%)	23	(85%)	16	(80%)	0.71[2]
Antenatal corticosteroids, n (%)	27	(100%)	20	(100%)	1.00[2]
Full course of antenatal corticosteroids, n (%)	18	(67%)	13	(65%)	1.00[2]
Patent ductus arteriosus, n (%)	26	(96%)	15	(75%)	0.07[2]
Surgical ligation of ductus arteriosus, n (%)	13	(48%)	6	(30%)	0.24[2]
Inclusion site - Linköping/Stockholm, n/n (%/%)	18/9	(67%/33%)	8/12	(40%/60%)	0.08[2]

Statistics: [1]Student's *t*-test, [2]Fisher's exact test, [3]Mann-Whitney *U* test. IQR = interquartile range, SD = standard deviation.

Table S3: Maternal and infant characteristics and *L. reuteri* abundance in the *L. reuteri*-supplemented group at one week of age.

Abundance (expressed as *L. reuteri* bacteria per 1 g wet faeces (median (IQR)) of *L. reuteri* in faeces of *L. reuteri*-supplemented infants, who did not (No) or did (Yes) have the indicated characteristic.

	**No**		**Yes**		
	***L. reuteri* levels Median (IQR)**	***n***	***L. reuteri* levels Median (IQR)**	***n***	***p* value[1]**
Born in gestational weeks 23-25	1.77×10^7 (1.18×10^6 - 7.53×10^7)	21	2.24×10^7 (3.94×10^6 - 1.18×10^8)	35	0.33
Small for gestational age (weight < 2 SD)	2.24×10^7 (3.43×10^6 - 8.77×10^7)	39	1.77×10^7 (1.86×10^6 - 1.21×10^8)	17	0.78
Female	1.61×10^7 (2.83×10^6 - 8.15×10^7)	24	3.54×10^7 (2.07×10^6 - 1.23×10^8)	32	0.58
Infant from multiple pregnancy	1.77×10^7 (3.26×10^6 - 7.53×10^7)	37	2.02×10^7 (1.69×10^6 - 1.34×10^8)	19	0.66
Caesarean section	5.34×10^7 (2.05×10^6 - 1.03×10^8)	14	1.75×10^7 (2.42×10^6 - 1.10×10^8)	42	0.69
Chorioamnionitis	1.75×10^7 (2.98×10^6 - 1.03×10^8)	40	3.19×10^7 (1.69×10^6 - 1.18×10^8)	16	0.81
Preeclampsia	2.24×10^7 (2.70×10^6 - 1.18×10^8)	51	3.59×10^6 (0 - 4.28×10^6)	5	0.07
Preterm premature rupture of membranes	7.13×10^6 (1.28×10^6 - 5.40×10^7)	35	6.26×10^7 (2.91×10^7 - 1.21×10^8)	21	**0.02**
Maternal smoking	1.75×10^7 (1.78×10^6 - 1.16×10^8)	52	4.89×10^7 (2.97×10^7 - 7.57×10^7)	4	0.47
Maternal antibiotics	1.72×10^7 (1.04×10^6 - 1.33×10^8)	21	2.24×10^7 (2.70×10^6 - 8.77×10^7)	35	0.87
Antenatal corticosteroids	1.72×10^7 (1.72×10^7 - 1.72×10^7)	1	2.02×10^7 (2.00×10^6 - 1.14×10^8)	55	0.93
Full course of antenatal corticosteroids	6.32×10^7 (1.72×10^7 - 1.20×10^8)	17	8.29×10^6 (1.11×10^6 - 6.39×10^7)	39	0.053
Included in Stockholm	1.72×10^7 (4.61×10^6 - 5.40×10^7)	19	2.91×10^7 (1.04×10^6 - 1.28×10^8)	37	0.97

Start of probiotic supplementation within 72 h[a]	4.42×10^7 (2.74×10^7 - 1.02×10^8)	7	1.61×10^7 (1.78×10^6 - 1.03×10^8)	48	0.30

[a]Data on whether probiotic supplementation was started within 72 hours after birth are missing for one infant with an *L. reuteri*-positive faecal sample. Statistics: [1]Mann-Whitney *U* test. IQR = interquartile range, NA = not applicable.

Table S4: Antibiotic treatment and *L. reuteri* prevalence in faeces of the *L. reuteri*-supplemented group.

Antibiotic treatment rates (number (%) for the indicated week) of *L. reuteri*-supplemented infants who had faeces positive (= *L. reuteri*-colonised) or negative (= non-colonised) for probiotic *L. reuteri* at one (**a**), two (**b**), three (**c**) and four (**d**) weeks of age.

**a: Week 1**					
	***L. reuteri*-colonised (*n* = 48)**		**Non-colonised (*n* = 8)**		***p* value[1]**
**Aminoglycoside**	48	(100%)	8	(100%)	1.00
**Ampicillin**	0	(0%)	0	(0%)	1.00
**Benzylpenicillin**	47	(98%)	8	(100%)	1.00
**Carbapenem**	6	(12%)	1	(12%)	1.00
**Cephalosporin**	2	(4%)	0	(0%)	1.00
**Cloxacillin**	0	(0%)	0	(0%)	1.00
**Macrolide**	2	(4%)	1	(12%)	1.00
**Metronidazole**	0	(0%)	1	(12%)	1.00
**Vancomycin**	1	(2%)	1	(12%)	1.00
**Any antibiotic**	**48**	**(100%)**	**8**	**(100%)**	**1.00**

Statistics: [1]Fisher's exact test with Benjamini-Hochberg correction.

**b: Week 2**					
	***L. reuteri*-colonised (*n* = 53)**		**Non-colonised (*n* = 2)**		***p* value[1]**
**Aminoglycoside**	37	(70%)	1	(50%)	1.00
**Ampicillin**	1	(2%)	0	(0%)	1.00
**Benzylpenicillin**	26	(49%)	0	(0%)	1.00
**Carbapenem**	9	(17%)	1	(50%)	1.00
**Cephalosporin**	6	(11%)	0	(0%)	1.00
**Cloxacillin**	0	(0%)	0	(0%)	1.00
**Macrolide**	6	(11%)	0	(0%)	1.00
**Metronidazole**	2	(4%)	1	(50%)	1.00
**Vancomycin**	9	(17%)	1	(50%)	1.00
**Any antibiotic**	**44**	**(83%)**	**2**	**(100%)**	**1.00**

Statistics: [1]Fisher's exact test with Benjamini-Hochberg correction.

**c: Week 3**					
	***L. reuteri*-colonised (*n* = 47)**		**Non-colonised (*n* = 4)**		***p* value[1]**
**Aminoglycoside**	21	(45%)	0	(0%)	0.65
**Ampicillin**	0	(0%)	0	(0%)	1.00
**Benzylpenicillin**	0	(0%)	0	(0%)	1.00
**Carbapenem**	5	(11%)	3	(75%)	0.10
**Cephalosporin**	7	(15%)	0	(0%)	1.00
**Cloxacillin**	8	(17%)	0	(0%)	1.00
**Macrolide**	5	(11%)	1	(25%)	1.00
**Metronidazole**	2	(4%)	0	(0%)	1.00
**Vancomycin**	13	(28%)	0	(0%)	1.00
**Any antibiotic**	**32**	**(68%)**	**3**	**(75%)**	**1.00**

Statistics: [1]Fisher's exact test with Benjamini-Hochberg correction.

**d: Week 4**					
	***L. reuteri*-colonised (*n* = 52)**		**Non-colonised (*n* = 1)**		***p* value[1]**
**Aminoglycoside**	20	(38%)	0	(0%)	1.00
**Ampicillin**	0	(0%)	0	(0%)	1.00
**Benzylpenicillin**	3	(6%)	0	(0%)	1.00
**Carbapenem**	11	(21%)	1	(100%)	1.00
**Cephalosporin**	8	(15%)	0	(0%)	1.00
**Cloxacillin**	5	(10%)	1	(100%)	1.00
**Macrolide**	7	(13%)	0	(0%)	1.00
**Metronidazole**	6	(12%)	0	(0%)	1.00
**Vancomycin**	22	(42%)	1	(100%)	1.00
**Any antibiotic**	**37**	**(71%)**	**1**	**(100%)**	**1.00**

Statistics: [1]Fisher's exact test with Benjamini-Hochberg correction.

Table S5: Antibiotic treatment and *L. reuteri* abundance in faeces of the *L. reuteri*-supplemented group.

Abundance (expressed as *L. reuteri* bacteria per 1 g faeces (median (IQR)) of *L. reuteri* in faeces of *L. reuteri*-supplemented infants, who were not (AB -) or were (AB +) treated with antibiotics for one to seven days in the first (**a**), second (**b**), third (**c**), and fourth (**d**) week of life.

**a: Week 1**					
	**AB -**		**AB +**		
**Antibiotic**	***L. reuteri* levels Median (IQR)**	***n***	***L. reuteri* levels Median (IQR)**	***n***	***p* value[1]**
**Aminoglycoside**	NA	0	1.90×10^7 (2.07×10^6 - 1.14×10^8)	56	NA
**Ampicillin**	1.90×10^7 (2.07×10^6 - 1.14×10^8)	56	NA	0	NA
**Benzylpenicillin**	4.68×10^5 (4.68×10^5 - 4.68×10^5)	1	2.02×10^7 (2.70×10^6 - 1.14×10^8)	55	0.42
**Carbapenem**	2.24×10^7 (3.26×10^6 - 1.13×10^8)	49	4.28×10^6 (8.24×10^5 - 6.25×10^7)	7	0.42
**Cephalosporin**	2.13×10^7 (1.93×10^6 - 1.14×10^8)	54	1.04×10^7 (6.99×10^6 - 1.38×10^7)	2	0.68
**Cloxacillin**	1.90×10^7 (2.07×10^6 - 1.14×10^8)	56	NA	0	NA
**Macrolide**	2.24×10^7 (2.14×10^6 - 1.15×10^8)	53	3.26×10^6 (1.63×10^6 - 5.20×10^6)	3	0.42
**Metronidazole**	2.02×10^7 (2.70×10^6 - 1.14×10^8)	55	0 (0 - 0)	1	0.42
**Vancomycin**	1.90×10^7 (2.42×10^6 - 1.10×10^8)	54	6.00×10^7 (3.00×10^7 - 9.00×10^7)	2	0.72
**Any antibiotic**	NA	0	1.90×10^7 (2.07×10^6 - 1.14×10^8)	56	NA

Statistics: [1]Mann-Whitney *U* test with Benjamini-Hochberg correction. AB = antibiotics, IQR = interquartile range, NA = not applicable.

b: Week 2					
	AB -		AB +		
**Antibiotic**	***L. reuteri* levels Median (IQR)**	***n***	***L. reuteri* levels Median (IQR)**	***n***	***p* value[1]**
**Aminoglycoside**	2.35×10^7 (6.94×10^6 - 9.14×10^7)	17	3.08×10^7 (1.17×10^7 - 7.26×10^7)	38	0.94
**Ampicillin**	3.06×10^7 (7.86×10^6 - 8.20×10^7)	54	2.42×10^8 (2.42×10^8 - 2.42×10^8)	1	0.28
**Benzylpenicillin**	3.09×10^7 (7.13×10^6 - 9.14×10^7)	29	2.93×10^7 (9.91×10^6 - 5.68×10^7)	26	0.94
**Carbapenem**	4.04×10^7 (1.12×10^7 - 8.52×10^7)	45	1.54×10^7 (2.25×10^6 - 3.08×10^7)	10	0.28
**Cephalosporin**	3.09×10^7 (7.36×10^6 - 8.52×10^7)	49	2.56×10^7 (1.57×10^7 - 3.80×10^7)	6	0.94
**Cloxacillin**	3.08×10^7 (8.37×10^6 - 8.44×10^7)	55	NA	0	NA
**Macrolide**	3.36×10^7 (1.12×10^7 - 8.52×10^7)	49	7.78×10^6 (4.78×10^6 - 1.11×10^7)	6	0.28
**Metronidazole**	3.06×10^7 (8.88×10^6 - 8.41×10^7)	52	6.02×10^7 (3.01×10^7 - 8.91×10^7)	3	0.94
**Vancomycin**	3.36×10^7 (9.38×10^6 - 9.14×10^7)	45	2.23×10^7 (8.90×10^6 - 3.98×10^7)	10	0.61
**Any antibiotic**	8.37×10^7 (4.10×10^7 - 1.15×10^8)	9	2.92×10^7 (7.19×10^6 - 5.98×10^7)	46	0.28

Statistics: [1]Mann-Whitney *U* test with Benjamini-Hochberg correction. AB = antibiotics, IQR = interquartile range, NA = not applicable.

c: Week 3					
	AB -		AB +		
Antibiotic	*L. reuteri* levels Median (IQR)	*n*	*L. reuteri* levels Median (IQR)	*n*	*p* value[1]
**Aminoglycoside**	5.94×10^7 (5.80×10^6 - 7.30×10^7)	30	8.32×10^7 (2.40×10^7 - 1.50×10^8)	21	0.21
**Ampicillin**	6.76×10^7 (1.18×10^7 - 1.22×10^8)	51	NA	0	NA
**Benzylpenicillin**	6.76×10^7 (1.18×10^7 - 1.22×10^8)	51	NA	0	NA
**Carbapenem**	6.91×10^7 (3.38×10^7 - 1.44×10^8)	43	4.01×10^6 (0 - 9.57×10^6)	8	**0.002**
**Cephalosporin**	6.79×10^7 (2.31×10^7 - 1.38×10^8)	44	1.10×10^7 (3.51×10^6 - 5.46×10^7)	7	0.45
**Cloxacillin**	6.27×10^7 (1.66×10^7 - 9.97×10^7)	43	1.04×10^8 (8.28×10^6 - 1.48×10^8)	8	0.64
**Macrolide**	6.78×10^7 (2.05×10^7 - 1.40×10^8)	45	1.58×10^7 (2.51×10^6 - 5.70×10^7)	6	0.21
**Metronidazole**	6.78×10^7 (1.10×10^7 - 1.37×10^8)	49	3.72×10^7 (2.89×10^7 - 4.56×10^7)	2	0.64
**Vancomycin**	6.79×10^7 (2.14×10^7 - 1.27×10^8)	38	5.40×10^7 (1.10×10^7 - 1.07×10^8)	13	0.97
**Any antibiotic**	6.84×10^7 (4.96×10^7 - 8.09×10^7)	16	5.40×10^7 (7.46×10^6 - 1.44×10^8)	35	0.64

Statistics: [1]Mann-Whitney *U* test with Benjamini-Hochberg correction. AB = antibiotics, IQR = interquartile range, NA = not applicable.

**d: Week 4**					
	**AB -**		**AB +**		
**Antibiotic**	***L. reuteri* levels Median (IQR)**	***n***	***L. reuteri* levels Median (IQR)**	***n***	***p* value[1]**
**Aminoglycoside**	3.79×10^7 (4.85×10^6 - 6.33×10^7)	33	1.61×10^7 (4.80×10^6 - 3.18×10^7)	20	0.40
**Ampicillin**	2.91×10^7 (4.85×10^6 - 5.62×10^7)	53	NA	0	NA
**Benzylpenicillin**	2.92×10^7 (4.96×10^6 - 5.44×10^7)	50	3.90×10^6 (2.56×10^6 - 1.93×10^8)	3	0.70
**Carbapenem**	3.36×10^7 (1.21×10^7 - 6.87×10^7)	41	4.69×10^6 (3.72×10^5 - 1.19×10^7)	12	**0.027**
**Cephalosporin**	2.91×10^7 (4.53×10^6 - 6.87×10^7)	45	2.81×10^7 (8.90×10^6 - 3.47×10^7)	8	0.70
**Cloxacillin**	3.11×10^7 (5.00×10^6 - 5.98×10^7)	47	6.56×10^6 (1.89×10^6 - 2.17×10^7)	6	0.18
**Macrolide**	2.92×10^7 (4.87×10^6 - 6.15×10^7)	46	2.85×10^7 (4.81×10^6 - 3.52×10^7)	7	0.70
**Metronidazole**	3.11×10^7 (6.52×10^6 - 6.60×10^7)	47	3.57×10^6 (8.30×10^5 - 1.84×10^7)	6	0.14
**Vancomycin**	3.58×10^7 (1.24×10^7 - 6.56×10^7)	30	9.22×10^6 (2.32×10^6 - 3.92×10^7)	23	0.18
**Any antibiotic**	4.24×10^7 (1.59×10^7 - 8.64×10^7)	15	2.54×10^7 (4.48×10^6 - 4.19×10^7)	38	0.18

Statistics: [1]Mann-Whitney *U* test with Benjamini-Hochberg correction. AB = antibiotics, IQR = interquartile range, NA = not applicable.

Table S6: Maternal Lewis and secretor status and *L. reuteri* colonisation in the *L. reuteri*-supplemented group.

Prevalence (**a, b**) and abundance (**c, d**) of *L. reuteri* in faeces of exclusively mother's own milk-fed *L. reuteri*-supplemented infants based on their mother's Lewis and secretor status. Prevalence is expressed as the number of infants with a faecal sample positive for *L. reuteri* at three weeks of age and the total number of infants who were exclusively fed their mother's own milk and whose mother is Lewis-positive (Le +) or Lewis-negative (Le -) (**a**), and secretor-positive (Se +) or secretor-negative (Se -) (**b**), respectively. *L. reuteri* abundance is expressed as bacteria per 1 g wet faeces of infants who were exclusively fed their mother's own milk and whose mother is Lewis-positive (Le +) or Lewis-negative (Le -) (**c**), and secretor-positive (Se +) or secretor-negative (Se -) (**d**), respectively.

**a: Lewis status and *L. reuteri* prevalence**				
**Le -**		**Le +**		***p* value[1]**
3 / 3	(100%)	30 / 33	(91%)	1.00

Statistics: [1]Fisher's exact test, [2]Mann-Whitney *U* test.

**b: Secretor status and *L. reuteri* prevalence**				
**Se -**		**Se +**		***p* value[1]**
7 / 9	(78%)	26 / 27	(96%)	0.15

Statistics: [1]Fisher's exact test, [2]Mann-Whitney *U* test.

**c: Lewis status and *L. reuteri* abundance**		
**Le –** **(n = 3)**	**Le +** **(n = 33)**	***p* value[2]**
6.76×10^7 (4.40×10^7 - 7.54×10^7)	5.66×10^7 (8.53×10^6 - 1.07×10^8)	1.00

Statistics: [1]Fisher's exact test, [2]Mann-Whitney *U* test.

**d: Secretor status and *L. reuteri* abundance**		
**Se –** **(n = 9)**	**Se +** **(n = 27)**	***p* value[2]**
2.82×10^7 (1.75×10^6 - 6.22×10^7)	6.89×10^7 (2.22×10^7 - 1.06×10^8)	0.19

Statistics: [1]Fisher's exact test, [2]Mann-Whitney *U* test.

Table S7: Human milk oligosaccharides and *L. reuteri* prevalence in the *L. reuteri*-supplemented group.

Concentrations of human milk oligosaccharides (in µmol/l) in mother's milk (n = 31) collected at two weeks postpartum from mothers of exclusively mother's own milk-fed infants from the *L. reuteri*-supplemented group (n = 36) with a faecal sample positive (*L. reuteri*-colonised, n = 33) or negative (non-colonised, n = 3) for *L. reuteri* at three weeks of age.

		***L. reuteri*-colonised**		**Non-colonised**			
**Human milk oligosaccharide**		**Median (range)**	***n***	**Median (range)**	***n***	***p* value[1]**	**Adjusted *p* value[2]**
Disialyl-lacto-N-tetraose	(DSLNT)	620 (180 - 1570)	33	1040 (440 - 1550)	3	0.41	0.70
2′-Fucosyllactose	(2′FL)	5840 (0 - 9460)	33	0 (0 - 9700)	3	0.60	0.70
3′-Fucosyllactose	(3′FL)	1300 (110 - 5140)	33	1850 (390 - 5600)	3	0.51	0.70
Lacto-difucotetraose	(LDFT)	440 (0 - 2710)	33	0 (0 - 690)	3	0.36	0.70
Lacto-N-difucohexaose I	(LNDH I)	910 (0 - 3020)	33	0 (0 - 1700)	3	0.60	0.70
Lacto-N-fucopentaose I	(LNFP I)	1040 (0 - 5270)	33	0 (0 - 5660)	3	0.60	0.70
Lacto-N-fucopentaose II	(LNFP II)	380 (0 - 3040)	33	1350 (280 - 3140)	3	0.20	0.59
Lacto-N-fucopentaose III	(LNFP III)	350 (90 - 850)	33	430 (270 - 760)	3	0.47	0.70
Lacto-N-neotetraose	(LNnT)	190 (0 - 600)	33	140 (110 - 400)	3	0.89	0.89
Lacto-N-tetraose	(LNT)	2050 (650 - 4970)	33	4340 (4170 - 4970)	3	**0.01**	0.16
Sialyl-lacto-N-tetraose a	(LSTa)	10 (0 - 50)	33	20 (10 - 30)	3	0.10	0.39
Sialyl-lacto-N-tetraose b	(LSTb)	70 (10 - 220)	33	120 (50 - 120)	3	0.37	0.70
Sialyl-lacto-N-neotetraose c	(LSTc)	120 (30 - 430)	33	210 (190 - 240)	3	0.06	0.31
3′-Sialyllactose	(3′SL)	330 (180 - 520)	33	370 (180 - 400)	3	0.84	0.89
6′-Sialyllactose	(6′SL)	1140 (350 - 1890)	33	1680 (1480 - 1800)	3	**0.03**	0.24

Statistics: [1]Mann-Whitney *U* test, [2]Benjamini-Hochberg correction.
